# Effects of Lidocaine Alone Versus Lidocaine–Dexmedetomidine Infusion on Pulmonary Gas Exchange and Respiratory Mechanics During Isoflurane Anesthesia in Horses

**DOI:** 10.3390/vetsci12111089

**Published:** 2025-11-16

**Authors:** Ludovica Chiavaccini, Raiane A. Moura, Tatiana Moreira Batista P. R. Azevedo, Chiara De Gennaro, Enzo Vettorato, Marta Romano, Diego A. Portela

**Affiliations:** 1 Department of Comparative, Diagnostic & Population Medicine, College of Veterinary Medicine, University of Florida, 1945 SW 16th Ave Room V2-117, Gainesville, FL 32608, USA; moreirab@uoguelph.ca (T.M.B.P.R.A.); cdegennaro@ufl.edu (C.D.G.); evettorato@ufl.edu (E.V.); marta.romano@ufl.edu (M.R.); dportela@ufl.edu (D.A.P.); 2 Department of Large Animal Clinical Sciences, College of Veterinary Medicine, University of Florida, 2015 SW 16th Ave, Gainesville, FL 32608, USA; mouraraiane@ufl.edu

**Keywords:** dexmedetomidine, F-shunt, horse, oxygenation, ventilation

## Abstract

Dexmedetomidine improves pulmonary function in dogs and humans, but evidence in horses is scarce. This study evaluated dexmedetomidine infusion on oxygenation and respiratory mechanics in anesthetized horses. Twenty horses undergoing elective surgery were included in a prospective, non-randomized, observational study. Horses received either lidocaine alone (1.3 mg/kg over 15 min, then 3 mg/kg/hour; LIDO) or combined with dexmedetomidine (1.75 μg/kg over 15 min, then 1.75 μg/kg/hour; DL). Respiratory mechanics, gas exchange, and cardiovascular variables were recorded at baseline, post-loading, and after 30, 60, and 90 min. Data were analyzed using mixed-effects linear models with horse as a random effect and time and treatment and their interaction as fixed effects (*p* ≤ 0.05). Peak inspiratory pressure increased over time with both treatments but was lower with DL at 90 min (−1.26 mmHg, *p* = 0.046). There was no evidence that arterial oxygen pressure or oxygenation ratio improved over time with DL (*p* > 0.75). Shunt fraction did not significantly change over time or between treatments (Wald χ^2^ = 4.77, *p* = 0.85). Heart rate with DL decreased from baseline (*p* ≤ 0.001) but remained higher than LIDO overall (*p* = 0.001). Dexmedetomidine infusion showed no benefit on oxygenation or respiratory mechanics in anesthetized horses.

## Simple Summary

Horses undergoing general anesthesia are at risk of low blood oxygen levels, which can lead to poor recovery and muscle or nerve damage. This study aimed to investigate whether giving a continuous dose of a sedative drug called dexmedetomidine during anesthesia could improve breathing, oxygen levels, and heart function in healthy horses. Twenty horses undergoing routine surgery were included, with half receiving dexmedetomidine in addition to lidocaine infusion and the other half receiving lidocaine alone. The researchers measured gas exchange, respiratory system function, and other physiologic variables at several time points during anesthesia. The results showed that while oxygen levels and lung function were similar between the two groups, horses receiving dexmedetomidine required slightly less anesthetic gas and developed lower pressures in the lungs during breathing. Heart rate changes were consistent with the known effects of dexmedetomidine, and blood pressure remained stable in all horses. These findings suggest that dexmedetomidine’s ability to improve ventilatory mechanics remains controversial and probably clinically trivial.

## 1. Introduction

Despite advancements in anesthetic techniques, monitoring modalities, and pharmacological agents, perianesthetic mortality in horses remains significantly higher than in small animal species [1,2]. Yet relatively few studies have focused specifically on perianesthetic complications. Among these, hypoxemia—defined as an arterial partial pressure of oxygen (PaO_2_) < 80 mmHg (10.7 kPa)—remains a concern due to its potential to compromise oxygen delivery to peripheral tissues, predisposing to the development of perianesthetic myopathy and neuropathy. Recent findings have linked intra-anesthetic hypoxemia to poor recovery quality in horses [3], reinforcing the clinical importance of timely detection and prevention. In a single-center study, hypoxemia was observed in 4% of horses undergoing elective procedures [4], underscoring its relevance even outside of emergency settings.

Over the past decade, intravenous administration of α_2_-adrenoceptor agonists as part of balanced anesthesia protocols has gained attention in equine practice, not only for their sedative properties and ability to enhance recovery quality [5], but also for their MAC-sparing, analgesic, and hemodynamic-modulating effects [6,7,8]. In Europe and the U.S., xylazine, detomidine, and romifidine are the only α_2_-agonists approved for use in horses. Although medetomidine and dexmedetomidine are licensed exclusively for small animal use, both have been experimentally and clinically investigated in equids [9,10,11].

Dexmedetomidine, the pharmacologically active enantiomer of medetomidine, is characterized by high clearance, a short terminal half-life, and minimal accumulation, making it well-suited for continuous infusion protocols [10,12]. In addition to its sedative and analgesic properties, dexmedetomidine has shown promise for its anti-inflammatory and organ-protective effects, particularly in ischemia–reperfusion injury models in both human and equine tissues [13,14,15,16,17]. In medicine, dexmedetomidine infusion has been associated with improved oxygenation, decreased intrapulmonary shunt, and enhanced pulmonary compliance [18,19]. Similarly, in anesthetized dogs, a constant rate intravenous infusion (CRI) of 1 μg/kg/hour of dexmedetomidine improved PaO_2_ and respiratory compliance and reduced airway resistance and shunt fraction (F-shunt) [20]. Although dexmedetomidine appeared to confer a protective effect against hypoxemia in a cohort of over 700 horses [4], the small number of cases receiving a dexmedetomidine CRI led to its exclusion from the final analysis.

The objective of this study was to assess the impact of a low-dose dexmedetomidine CRI on oxygenation, respiratory system mechanics, and hemodynamic variables in systemically healthy horses anesthetized with isoflurane and a CRI of lidocaine. We hypothesized that the adjunct of dexmedetomidine would improve respiratory function, as evidenced by better oxygenation, decreased F-shunt, and improved respiratory system compliance.

## 2. Materials and Methods

### 2.1. Study Design

A prospective, observational, non-randomized study with group matching was conducted at the University of Florida, College of Veterinary Medicine. Ethical approval from the Institutional Animal Care and Use Committee (IACUC) was waived prior to the start of data collection, as the study was purely observational and no horse would receive any unique treatment because of the study (IACUC202200000265).

### 2.2. Animals

Horses undergoing elective inhalant isoflurane anesthesia in dorsal recumbency and classified as American Society of Anesthesiologists (ASA) physical status I or II were eligible for inclusion if they were scheduled to receive a lidocaine infusion alone or in combination with dexmedetomidine. Horses younger than 1 year old, pregnant mares, and horses with known or suspected respiratory disease were excluded. Ponies weighing less than 250 kg were also excluded. The group assignment was not randomized but based on the anesthetic protocol selected by the attending clinician. Based on the findings of Di Bella et al. [20] in dogs, along with preliminary data from five horses anesthetized with isoflurane and receiving a lidocaine infusion, we calculated the required sample size to detect a 30% reduction in the F-shunt and a 30% improvement of the arterial partial pressure over fraction inspired oxygen (PaO_2_:FiO_2_) ratio at 30 min after initiating a dexmedetomidine infusion. Assuming a power of 0.8 and an α level of 0.01 (to account for repeated measurements), we determined that a minimum of eight horses per group was necessary. If any horse experienced a PaO_2_ below 100 mmHg during the study, they were withdrawn and treated at the clinician’s discretion. Data from these horses were included up until the point of withdrawal, and a replacement horse was enrolled to maintain the group size.

### 2.3. Anesthesia Management and Monitoring

Grain, but not hay, was withheld for 8 h prior to anesthesia, and water was provided ad libitum. A 14-gauge catheter was placed in a jugular vein for intravenous (IV) fluid and drug administration. Sedation was achieved with xylazine (0.5 mg/kg Rompun^®^, Dechra, Northwich, UK) and butorphanol (0.02 mg/kg; Torbugesic; Zoetis Inc., Parsippany, NJ, USA). Flunixin meglumine (1.1 mg/kg; Banamine, Merck Animal Health, Madison, NJ, USA), potassium penicillin, and gentamicin (at standard dosages) were administered IV at the clinician’s discretion.

General anesthesia was induced with midazolam (0.05 mg/kg; Midazolam Inj.; Avet Pharmaceuticals Inc., Mahwah, NJ, USA) and ketamine (2.2 mg/kg; Ketaset; Zoetis Inc., NJ, USA). Orotracheal intubation was performed with a 26 mm internal diameter silicone cuffed endotracheal tube. Horses were hoisted and positioned in dorsal recumbency on a padded surgical table. Anesthesia was maintained with isoflurane in oxygen, as determined by the attending clinician.

After positioning in dorsal recumbency, horses were connected to a piston-driven ventilator (Tafonius; Hallowell EMC, Pittsfield, MA, USA), and volume-controlled ventilation was initiated with a tidal volume (V_T_) of 10–15 mL/kg, respiratory rate (*f*R) of 5–6 breaths/minute, inspiratory time of 3 s, an inspiratory-to-expiratory (I:E) ratio of 1:2, positive end-expiratory pressure (PEEP) of 5 cmH_2_O, maintaining peak inspiratory pressure (PIP) ≤ 30 cmH_2_O. Respiratory rate was adjusted to maintain the end-tidal carbon dioxide tension (Pe′CO_2_) between 40 and 50 mmHg (5.3–6.6 kPa). Plateau pressure (P_plat_) was calculated by adding an end-inspiratory pause of 30%.

Physiologic monitoring included heart rate (HR; base-apex, lead I ECG), hemoglobin oxygen saturation (SpO_2_), end-tidal isoflurane concentration (Fe′Iso), fraction of inspired oxygen (FiO_2_), and esophageal temperature, recorded continuously using a multiparameter monitor (Solomon; Vetronic Services Ltd., Newton Abbot, UK). Arterial blood pressure was monitored via a 20-gauge over-the-needle catheter placed in the facial or transverse facial artery and connected to a pressure transducer (Deltran^®^; Utah Medical Products Inc., Midvale, UT, USA) through a non-compliant extension line filled with heparinized saline (Heparin 10,000 USP units/mL; Meitheal Pharmaceutical, Chicago, IL, USA). The transducer was zeroed at atmospheric pressure and leveled at the right atrium, approximated at the shoulder joint.

Mainstream Pe′CO_2_ and spirometric variables were measured using an adult CO_2_/flow sensor (Respironics Novametrix LLC., Wallingford, CT, USA) attached to a respiratory profile monitor (NM3; Phillips Respironics, Respironics Novametrix LLC., Wallingford, CT, USA). A 3D-printed flow partitioning device, described by Schramel et al. [21] and previously validated in horses [22], connected the adult human-size sensor to the equine endotracheal tube. Only one of the four flow-partitioned outputs was connected to the NM3 monitor. The mainstream capnograph was zeroed at room air, and the pneumotachograph’s accuracy was regularly verified with a 3L calibration syringe (Adjustable 3L Calibration Syringe; A-M Systems Inc., Sequim, WA, USA). All measured volumes were multiplied by a factor of four to account for the flow partitioning.

Lactated Ringer’s solution (5 mL/kg/hour) was infused IV throughout anesthesia. If mean arterial pressure (MAP) decreased below 70 mmHg, a dobutamine infusion (0.5–2 µg/kg/minute; Dobutamine injection USP, Hospira Inc., Lake Forest, IL, USA) was initiated and titrated at the clinician’s discretion. The administered dose rate was recorded.

### 2.4. Study Protocol

Only horses receiving a CRI of lidocaine (Lidocaine Hydrochloride Injection 2%; Vedco Inc., Saint Joseph, MO, USA; group LIDO), with or without a concurrent dexmedetomidine CRI (Dexdomitor; Zoetis Inc., NJ, USA; group DL), were enrolled in the study. The doses of lidocaine and dexmedetomidine used corresponded to common protocols employed at the University of Florida, based on published literature. Specifically, for lidocaine, a loading dose of 1.3 mg/kg over 15 min was followed by a maintenance infusion of 3 mg/kg/hour [23]. A dexmedetomidine loading dose of 1.75 μg/kg over 15 min was followed by a maintenance infusion of 1.75 μg/kg/hour [9]. The administration of the drugs under study and the related monitoring of parameters took place once the horses were under general anesthesia and had reached a stable anesthetic plane. Infusions were initiated after anesthetic equilibration and within 30 min of connecting the horse to the anesthesia machine. Respiratory variables were recorded at five time points: five minutes before drug administration (BASELINE), at the end of the loading dose (BOLUS), and at 30, 60, and 90 min after the start of the infusion. Arterial blood samples were taken anaerobically at the same time points and analyzed using a handheld blood analyzer (i-STAT 1 Analyzer; Abbott Point of Care Inc., Princeton, NJ, USA, and i-STAT CG8 test cartridges; Abbott Point of Care Inc., Princeton, NJ, USA). Results were not adjusted for body temperature. Data from the arterial blood gas analysis—including pH, PaO_2_, PaCO_2_, and hemoglobin concentration—were recorded in a dedicated Excel spreadsheet (Microsoft^®^ Excel for Mac, Version 16.92; Microsoft, Redmond, WA, USA) for analysis. The atmospheric pressure (P_Atm_) was measured by consulting the local weather station and recorded at each time point.

### 2.5. Data Elaboration

Raw data were extracted at a 200 Hz sampling rate from the NM3 monitor software (version 2.2) and analyzed offline using the ICU-Lab 2.7 Software Package (KleisTEK^®^ Advanced Electronic Systems, Bari, Italy). Individual breaths were manually selected based on their modeled volumetric capnography (VCap) curves and exported to a Microsoft^®^ Excel for Mac spreadsheet (Version 16.92). The Tusman model (7-parameter model) implemented in the ICU-Lab Software Package was used for analysis. At each time point, values from 10 representative breaths were averaged. Extracted variables included expiratory tidal volume (V_T_), Pe′CO_2_, mixed expired CO_2_ pressure (P_Ē_CO_2_), PIP, plateau pressure (P_plat_), and PEEP.

Respiratory variables and arterial blood gas results were further analyzed offline using custom Excel spreadsheets with previously validated equations. Minute ventilation (V̇e) was calculated by multiplying V_T_ by *f*R, normalized to body weight, and reported in mL/minute/kg. Dynamic respiratory system compliance (C_dyn_) was determined as V_T_/(PIP—PEEP) and normalized to body weight. Quasi-static respiratory system compliance (C_qstat_) was similarly calculated as V_T_/(P_plat_—PEEP) and normalized to body weight.

The estimated F-shunt (%) was calculated using the validated formula described by Araos et al. [24] and previously applied in horses [25,26]:

(1) $$Fshunt=\frac{C_{c\prime }O_{2}-C_{a}O_{2}}{CC_{c\prime }O_{2}-C_{a}O_{2}+3.5\;mL/dL}\times 100$$

where C_c′_O_2_ is the capillary content of oxygen and C_a_O_2_ is the arterial oxygen content.

V_D_/V_T_ ratio was calculated using the Enghoff equation as,

(2) $$\frac{V_{D}}{V_{T}}=\frac{P_{a}{\mathrm{CO}}_{2}-P_{\bar{E}}{\mathrm{CO}}_{2}}{P_{a}{\mathrm{CO}}_{2}}$$

The arterial to end-tidal CO_2_ difference (Pa-Pe’CO_2_) was calculated for each time point.

### 2.6. Statistical Analysis

Data were processed and analyzed using Microsoft^®^ Excel for Mac version 16.92 (Microsoft Corp., Redmond, WA, USA) and Stata/BE 17.0 for Mac (StataCorp LLC., College Station, TX, USA). Continuous data were assessed for normality using the Shapiro–Wilk test and visually inspected with histograms and Q-Q plots (qnorm function in Stata). Data were reported as mean ± standard deviation (SD) if normally distributed and median [interquartile range (Q1, Q3)] if not normally distributed. Normally distributed data were compared at baseline using Student’s *t*-test, while nonparametric data were compared using the Wilcoxon rank sum test. The HR, *f*R, MAP, P_plat_, SpO_2_, Pa-Pe’CO_2_, and V_D_/V_T_ did not meet the normality assumption and were rank transformed before further analysis [27]. All data were analyzed using a mixed-effect linear model using the horse as a random effect and time, treatment, and their interaction as fixed effects. Assumptions of the homoskedasticity and normality distribution of the residuals were graphically checked.

To identify distinct subgroups of horses with similar trajectories in respiratory mechanics and gas exchange parameters, group-based trajectory modeling was performed using the traj function in Stata, following the approach described by Haviland et al. [28]. This method fits polynomial functions (linear, quadratic, and cubic) to longitudinal data to capture differing patterns of physiological change over time—such as variations in pulmonary compliance, F-shunt, and PaO_2_:FiO_2_ ratio. Model development followed a stepwise, data-driven strategy. All groups were initially modeled with linear terms. Higher-order terms (quadratic, then cubic) were tested incrementally for each group and retained only when they significantly improved model fit, as evaluated by the Bayesian Information Criterion (BIC). This process yielded a model in which each group’s trajectory was described by the polynomial order best fitting its progression, allowing for different shapes across groups. Significance was set at *p* ≤ 0.05 throughout.

## 3. Results

**Figure 1:**
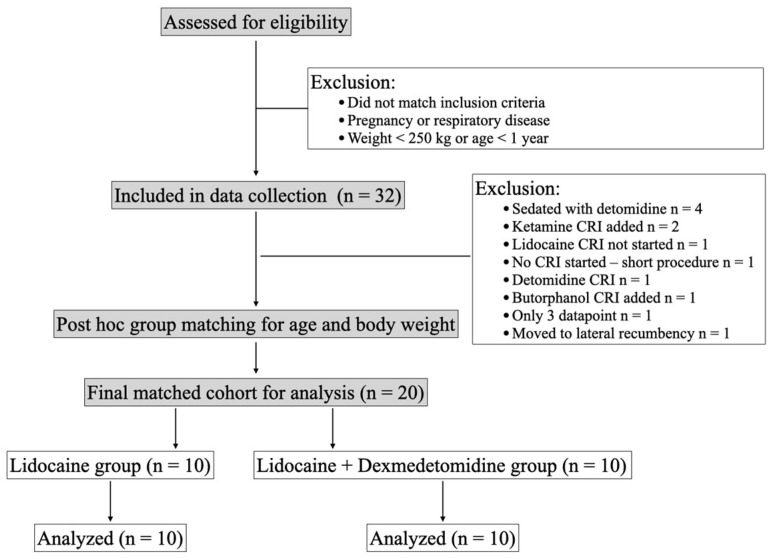
Data collection and exclusion criteria.

A total of 32 horses were included in the data collection, from which 20 horses were ultimately selected for the final analysis (Figure 1). Horses were matched post hoc for age and body weight to reduce group imbalance and in consideration of these variables’ possible effects on respiratory mechanics and gas exchange [4,29]. Demographic data are reported in Table 1. Bodyweight (median difference = 6 kg, 95% confidence interval (CI) −60–150, z = 0.30, *p* = 0.78) and age (mean difference = 3.1 ± 3.50 years, 95% CI −10.4–4.2, *p* = 0.39) were not significantly different between the two groups. Likewise, there was no statistically significant difference in the mean atmospheric pressure at the beginning of the study (mean difference = −1.47 mmHg, 95% CI −4.07–1.07, *p* = 0.24). For inhaled anesthetics, there was a significant interaction between time and treatment, with horses in the DL group recording a significantly lower Fe’Iso at 60 min post-infusion than baseline (coefficient −0.18%, SE 0.08, 95% CI −0.33–−0.038, *p* = 0.014).

### 3.1. Respiratory System Mechanics

At baseline, there were no statistically significant differences between the LIDO and DL groups for any of the measured respiratory variables, including fR, V̇e, relative C_dyn_ and C_qstat_, PIP and P_plat_. Regardless of treatment, fR increased over time beginning 30 min after the loading dose (LR χ^2^ = 21.63, *p* < 0.001), although this change was not considered clinically relevant. The V̇e also increased over time in both groups and was significantly higher at 60 (coefficient 9.49 mL/minute/kg, SE 3.15, 95% CI 1 3.32–15.67, *p* = 0.003) and 90 min (coefficient 11.30 mL/minute/kg, SE 3.33, 95% CI 1 4.77–17.81, *p* = 0.001) of infusion, respectively. Peak inspiratory pressure increased over time in both groups (*p* ≤ 0.01) but was significantly lower in the DL group at 90 min (coefficient −1.26 mmHg, SE 0.64, 95% CI −2.51–−0.02, *p* = 0.046). No significant differences were observed in P_plat_ between groups or over time (Wald c^2^ = 4.93, *p* = 0.09). Dynamic compliance decreased at 30 (coefficient −0.05 mL/cmH_2_O/kg, SE 0.02, *p* = 0.028) and 90 min (coefficient −0.08 mL/cmH_2_O/kg, SE 0.02, *p* = 0.001) compared to baseline, with no significant effect of treatment. Trajectory analysis identified three distinct C_dyn_ patterns, with a small subgroup (5%) showing a progressive decrease over time, but treatment allocation did not influence group membership (*p* = 0.99). Similarly, C_qstat_ did not change significantly over time (Wald c^2^ = 15.75, *p* = 0.072), with 10% of subjects showing a decline that was not treatment related. Respiratory system mechanics results are summarized in Table 2.

### 3.2. Gas Exchange Parameters

At baseline, there were no statistically significant differences between treatment groups for PaO_2_ (LIDO: 336.4 ± 165.60 mmHg; DL: 362.7 ± 118.18 mmHg; *p* = 0.69) or PaO_2_:FiO_2_ ratio (LIDO: 366.83 ± 179.37 mmHg; DL: 399.86 ± 131.52 mmHg; *p* = 0.64). Both variables did not change over time without significant treatment effects (PaO_2_: Wald χ^2^ = 10.15, *p* = 0.34; PaO_2_:FiO_2_: Wald χ^2^ = 5.90, *p* = 0.75). F-shunt was also comparable at baseline between groups (LIDO: 17.31 ± 9.62%; DL: 15.93 ± 7.54%; *p* = 0.73), with no significant change over time or between treatments (Wald χ^2^ = 4.77, *p* = 0.85). Trajectory analysis of PaO_2_:FiO_2_ identified three subgroups: 26.2% of horses started below 200 and showed non-significant improvement; 43.8% remained stable between 300 and 400; and 29.9% remained >500 throughout. Similar trends were observed for F-shunt, with 34.6% of horses maintaining values < 10%, 37.3% between 15 and 20%, and 28.2% > 20%, without treatment effect on distribution. Arterial saturation of oxygen values did not differ over time or between groups (Wald χ^2^ = 7.47, *p* = 0.59).

Arterial CO_2_ partial pressure (*p* = 0.32) and Pe’CO_2_ (*p* = 0.69) were also not significantly different at baseline or over time (PaCO_2_: Wald χ^2^ = 7.85, *p* = 0.55; Pe’CO_2_: Wald χ^2^ = 16.81, *p* = 0.06). The Pa- Pe’CO_2_ did not differ between groups (Wald χ^2^ = 12.94, *p* = 0.16). A statistically significant difference was observed in Vd/Vt at baseline (*p* = 0.01), though no overall effect of treatment or time was detected (Wald χ^2^ = 10.54, *p* = 0.31). Trajectory analysis revealed three Vd/Vt patterns: in 54.8% of horses, it remained <0.35, 28.6% recorded a Vd/Vt between 0.35 and 0.45, and in 17.2%, it was >0.45. Full gas exchange data are presented in Table 3.

### 3.3. Cardiovascular Variables

Following the loading dose, HR in the DL group significantly decreased and remained lower than baseline throughout the study period (*p* ≤ 0.00. Regardless of treatment group, MAP increased significantly over time compared to baseline (LR χ^2^ = 13.77, *p* = 0.008). Horses in the DL group required in average higher doses of dobutamine (coefficient 0.50, SE 0.22, 95% CI 0.07–0.92, *p* = 0.002), with no differences between groups over time (LR χ^2^ = 5.16, *p* = 0.27). Cardiovascular variables over time are summarized in Table 4.

## 4. Discussion

This study found no evidence that the addition of a dexmedetomidine infusion to a lidocaine infusion improved respiratory mechanics or gas exchange in horses anesthetized with isoflurane and maintained in dorsal recumbency. These findings contrast with results reported in dogs undergoing ovariectomy under isoflurane general anesthesia and receiving 1 µg/kg of dexmedetomidine IV followed by a CRI at 1 µg/kg/hour by Di Bella et al. [20], where dexmedetomidine significantly decreased P_plat_ and F-shunt while increasing C_qstat_ and the PaO_2_:FiO_2_ ratio. Similarly, in humans, several studies have shown that dexmedetomidine infusions improve oxygenation and respiratory mechanics by increasing compliance, decreasing dead space, and lowering plateau pressures [18,19,30,31]. In contrast, studies in small ruminants have shown that dexmedetomidine administration can impair pulmonary function. For instance, Kutter et al. [32] observed significant reductions in C_dyn_, with increases in F-shunt and dead space ventilation following 2 µg/kg IV dexmedetomidine in sheep and goats—effects that persisted throughout the observation period. These interspecies differences are likely due to differing pulmonary responses to α_2_-adrenoreceptor agonists. In dogs and in humans, the respiratory benefits of α_2_-agonists are attributed to mild bronchodilation, improved hypoxic pulmonary vasoconstriction decreased by inhalant anesthetics, and better ventilation–perfusion matching [18,20,33]. Dexmedetomidine has also been proposed to enhance endogenous nitric oxide production, thereby reducing intrapulmonary shunt and improving arterial oxygenation [30]. Conversely, in sheep and goats, α2-agonists may induce bronchoconstriction, vasoconstriction, or both, possibly contributing to pulmonary congestion or edema [32]. This is supported by findings that α2-receptors in the trachea can mediate bronchospasm in sheep, as tracheal contraction occurs upon stimulation—an effect reversible by atipamezole but not by atropine [34,35]. In horses under anesthesia, the alveolar–arterial oxygen gradient increases rapidly post-induction and is not significantly improved by conventional positive pressure ventilation. This gradient reflects a combination of hypoventilation and ventilation/perfusion mismatch, with compression atelectasis—due to abdominal pressure on the lungs—playing a more prominent role than absorption atelectasis [36,37]. Unlike absorption atelectasis, which may be alleviated by reducing FiO_2_, compression atelectasis may require mechanical interventions such as reverse-Trendelenburg positioning [38]. In anesthetized humans, the normal range of F-shunt is typically considered to be 4–10% [30]. Shunt fraction in anesthetized horses varies significantly with body position, rising from ~1% while standing to 33% in dorsal recumbency [39]. These postural effects are likely major contributors to the hypoxemia commonly observed in equine anesthesia [29]. In the present study, F-shunt values ranged from 12% to 18%, improving over time but exhibiting wide individual variability, consistent with previous reports [26,29,36,38]. Unlike other studies, we chose to retain horses with larger F-shunt values in the analysis until an intervention became necessary. This decision probably contributed to the observed variability and decreased the statistical power of the analysis. Based on current data, we estimate that detecting a statistically significant improvement in F-shunt at 90 min post-infusion would require approximately 131 horses per group. Nonetheless, this individual variability is clinically meaningful, as supported by the trajectory analysis, which identified three distinct groups. One of these groups—comprising roughly 25–30% of the horses—consistently showed higher F-shunt values and lower C_dyn_ and C_qstat_. If confirmed in larger cohorts, this finding may represent an initial step toward identifying risk factors that predispose certain horses to perianesthetic hypoxemia. Additionally, the application of PEEP immediately after induction and connection to the anesthesia machine may have altered the distribution of ventilation, increased PaO_2_, and improved functional residual capacity, potentially masking intergroup differences, as corroborated by an overall lower than expected F-shunt [40,41].

While the HR and dobutamine requirements were overall higher in the DL group than the LIDO group, this was probably offset by the higher values recorded at baseline, before any infusion was initiated, and no difference between groups was observed over time.

This study has several limitations. First, the clinical nature of the work precluded a crossover design. Horses were not randomized to treatment groups; rather, anesthetic management was left to the discretion of the attending anesthesiologist. At our institution, different clinicians routinely use different anesthetic protocols based on personal preference. Cases in which the protocol required adjustment or was modified intraoperatively were excluded from analysis. Group selection primarily considered age and body weight, as these variables are known to influence respiratory parameters and F-shunt, thereby minimizing potential confounding effects. Additionally, a subset of horses participated in an unrelated experimental study involving jejunocecostomy. While it could be argued that opening the abdominal cavity might have reduced intra-abdominal pressure due to the sloped diaphragm and overlying intestinal contents [42], these horses did not exhibit lower F-shunt values or improved C_qstat_ during the procedure, suggesting the effect was clinically negligible, particularly given that they were healthy, with no gastrointestinal compromise. To maintain the Pe’CO_2_ between 40 and 50 mmHg (5.3–6.6 kPa), a progressive increase in respiratory rate was required. Similar increases in Pe’CO_2_ despite mechanical ventilation have been previously reported in healthy, isoflurane-anesthetized horses [43]. The increase in *f*R likely shortened expiratory time and decreased the I:E ratio, potentially causing air trapping or overinflation of the lungs. However, this effect was consistent across all treatment groups. Lastly, while F-shunt is a content-based index that estimates venous admixture without requiring mixed venous sampling, it assumes a fixed arterial-to-mixed venous oxygen content difference of 3.5 mL/dL. This simplification may fail to account for variations in tissue oxygen extraction due to changes in perfusion or metabolism. Nevertheless, the F-shunt has shown good agreement with the traditional Berggren equation for estimating venous admixture in horses [26].

## 5. Conclusions

In conclusion, while the addition of a constant-rate dexmedetomidine infusion to lidocaine reduced inhalant anesthetic requirements, it did not significantly improve F-shunt or ventilatory mechanics in mechanically ventilated, isoflurane-anesthetized horses in dorsal recumbency. The use of longitudinal trajectory analysis revealed a distinct subgroup of horses that consistently exhibited impaired gas exchange and reduced respiratory system compliance. This analytical approach may be instrumental in identifying individuals at greater risk of developing perianesthetic hypoxemia. Future studies with larger cohorts are warranted to validate these findings and refine the predictive power of trajectory-based models in equine anesthesia.

## Figures and Tables

**Table 1:** Demographic information of 20 healthy adult horses anesthetized with isoflurane and a of lidocaine (1.3 mg/kg loading dose, followed by 3 mg/kg/hour) with (DL) or without (LIDO) the addition of dexmedetomidine (1.75 µg/kg loading dose, followed by 1.75 µg/kg/hour). AQH America Quarter Horse; F, female; G, gelding; M, male; TB, Thoroughbred.

Variable	Horse									
	LIDO									
	#1	#2	#3	#4	#5	#6	#7	#8	#9	#10
**Age (years)**	21	17	15	14	6	3	10	3	4	5
**Sex**	G	F	F	F	F	F	F	G	G	G
**Breed**	AQH	Saddlehorse	AQH	AQH	TB	TB	TB	TB	TB	TB
**Procedure performed**	Elective laparotomy	Elective laparotomy	Elective laparotomy	Elective laparotomy	Elective laparotomy	Elective laparotomy	Elective laparotomy	Elective laparotomy	Elective laparotomy	Elective laparotomy
	**DL**									
	**#1**	**#2**	**#3**	**#4**	**#5**	**#6**	**#7**	**#8**	**#9**	**#10**
**Age (year)**	4	13	3	15	18	27	20	10	3	18
**Sex**	M	G	F	G	G	G	F	F	F	G
**Breed**	Paint	AQH	AQH	TB	Paint	Paint	Saddlehorse	Paint	AQH	AQH
**Procedure performed**	Cryptorchid castration	Carpus arthroscopy	Coronary wound debridement	Distal phalanx debridement	Partial phallectomy	Mass excision and cisplatin beads implantation	Elective laparotomy	Elective laparotomy	Cryptorchid castration	Mass excision and cisplatin beads implantation

**Table 2:** Respiratory system mechanics expressed as median (interquartile range) or mean ± standard deviation in 20 healthy adult horses anesthetized with isoflurane and a of lidocaine (1.3 mg/kg loading dose, followed by 3 mg/kg/hour) with (DL) or without (LIDO) the addition of dexmedetomidine (1.75 µg/kg loading dose, followed by 1.75 µg/kg/hour). C_dyn_, dynamic compliance; C_qstat_, quasi-static compliance; fR, respiratory rate; PIP, peak inspiratory pressure; P_plat_, plateau pressure; V̇e, minute ventilation.

Variable		Baseline	Bolus	30 min	60 min	90 min
*f*R (breaths/minute)	LIDO	5 (5, 6)	6 (5, 6) *	6 (5, 6) *	6 (5, 6) *	6 (5.5, 7) *
	DL	5 (5, 5)	5 (5, 6) *	5.5 (5, 7) *	6 (5, 6) *	6 (5, 6.5) *
V̇e (mL/minute/kg)	LIDO	83.01 ± 18.69	85.77 ± 17.00	88.69 ± 18.44	96.44 ± 19.66 *	98.96 ± 22.2 *
	DL	81.18 ± 9.61	84.46 ± 11.93	88.16 ± 21.84	85.95 ± 16.00 *	88.23 ± 14.93 *
C_dyn_ (mL/cmH_2_O/kg)	LIDO	0.70 ± 0.12	0.66 ± 0.10	0.65 ± 0.11 *	0.67 ± 0.12	0.66 ± 0.11 *
	DL	0.74 ± 0.21	0.74 ± 0.24	0.70 ± 0.23 *	0.70 ± 0.28	0.69 ± 0.15 *
C_qstat_ (mL/cmH_2_O/kg)	LIDO	0.81± 0.12	0.76 ± 0.09	0.75 ± 0.11	0.79 ± 0.10	0.79 ± 0.14
	DL	0.87 ± 0.20	0.90 ± 0.26	0.82 ± 0.27	0.80 ± 0.26	0.87 ± 0.24
PIP (cmH_2_O)	LIDO	26.97 ± 3.74	27.15 ± 3.62	28.04 ± 3.81 *	28.97± 3.34 *	28.61 ± 1.93 *
	DL	27.26 ± 4.24	27.30 ± 4.33	27.82 ± 4.54 *	28.11 ± 4.96 *	27.32 ± 3.31 *^,†^
P_plat_ (cmH_2_O)	LIDO	23.2 (21.5, 25.0)	23.1 (22.1, 25.0)	24.0 (22.8, 26.4)	25.1 (22.5, 26.0)	25.5 (20.8, 26.5)
	DL	22.7 21 (21, 26.5)	22.7 (21, 24.6)	24.0 (21.2, 27.5)	25.0 (22.0, 26.3)	22.5 (20.7, 25.7)

* significantly different from baseline (*p* ≤ 0.05). ^†^ significantly different between treatments (*p* ≤ 0.05).

**Table 3:** Gas exchange parameters expressed as median (interquartile range) or mean ± standard deviation in 20 healthy adult horses anesthetized with isoflurane and a of lidocaine (1.3 mg/kg loading dose, followed by 3 mg/kg/hour) with (DL) or without (LIDO) the addition of dexmedetomidine (1.75 µg/kg loading dose, followed by 1.75 µg/kg/hour). PaCO_2_, arterial partial pressure of carbon dioxide; PaO_2_, arterial partial pressure of oxygen; Pa-Pe’CO_2_, arterial to end-tidal carbon dioxide partial pressure difference; PaO_2_:FiO_2_, arterial partial pressure over fraction inspired oxygen ratio; Pe’CO_2_, end-tidal carbon dioxide partial pressure; SpO_2_, arterial saturation of oxygen measured by pulse oximetry; V_D_/V_T_, physiologic dead space.

Variable		Baseline	Bolus	30 min	60 min	90 min
PaO_2_ (mmHg)	LIDO	336.4 ± 165.6	338.3 ± 192.0	351.3 ± 169.1	367.5 ± 180.5	409.4 ± 142.4
	DL	362.7 ± 118.2	398.8 ± 142.8	377.2 ± 150.4	445.3 ± 96.3	449.0 ± 97.7
PaO_2_:FiO_2_	LIDO	366.83 ± 179.37	363.24 ± 208.25	373.64 ± 180.49	390.29 ± 192.09	433.16 ± 150.42
	DL	399.86 ± 131.52	424.47 ±151.32	396.70 ± 158.81	464.71 ± 98.66	468.21 ± 101.06
F-shunt (%)	LIDO	17.31 ± 9.62	17.79 ± 12.32	17.70 ± 10.55	16.58 ± 11.24	14.63 ± 9.28
	DL	15.93 ± 7.54	14.91 ± 8.73	16.5 ± 8.97	13.00 ± 7.36	12.77 ± 7.33
SpO_2_ (%)	LIDO	98 (97, 98)	98 (96, 99)	98 (97, 98)	97.5 (96, 98)	98 (97, 98)
	DL	97 (97, 98)	97 (95, 98)	96.5 (96, 98)	98 (96, 98)	98 (96, 98.5)
PaCO_2_ (mmHg)	LIDO	50.18 ± 5.40	49.41 ± 4.42	49.48 ± 5.46	49.66 ± 6.44	49.01 ± 5.39
	DL	53.29 ± 7.93	53.15 ± 8.67	55.75 ±10.87	54.04 ± 8.97	54.5 ± 8.52
PaCO_2_ (kPa)	LIDO	6.69 ± 0.72	6.59 ± 0.59	6.60 ± 0.73	6.62 ± 0.86	6.53 ± 0.72
	DL	7.10 ± 1.0	7.09 ± 1.16	7.43 ± 1.45	7.20 ± 1.20	7.27 ± 1.14
Pe’CO_2_ (mmHg)	LIDO	40.55 ±10.52	41.87 ± 7.03	38.75 ± 4.19	36.87 ± 4.36	38.94 ± 4.71
	DL	42.12 ± 6.12	43.59 ± 6.75	44.37 ± 7.16	44.09 ± 6.79	44.31 ± 7.11
Pe’CO_2_ (kPa)	LIDO	5.41 ± 1.40	5.58 ± 0.94	5.17 ± 0.56	4.92 ± 0.58	5.19 ± 0.63
	DL	5.62 ± 0.82	5.81 ± 0.90	5.92 ± 0.95	5.88 ± 0.91	5.91 ± 0.95
Pa-Pe’CO_2_ (mmHg)	LIDO	10.6 (7.1, 14.4)	7.6 (6.0, 10.0)	9.85 (8.6, 12.4)	11.4(9.9, 14.0)	10.4 (7.9, 13.0)
	DL	10.3 (8.6, 12.7)	9.6 (7.0, 12.8)	12.3 (8.1, 14.8)	9.9 (7.8, 13.4)	9.7 (8.4, 12.7)
Pa-Pe’CO_2_ (kPa)	LIDO	1.41 (0.95, 1.92)	1.01 (0.80,1.33)	1.31 (1.15, 1.65)	1.52 (1.32, 1.87)	1.39 (1.05, 1.73)
	DL	1.37 (1.15, 1.69)	1.28 (0.93, 1.71)	1.64 (1.08, 1.97)	1.32 (1.04,1.79)	1.29 (1.12, 1.69)
Vd/Vt	LIDO	0.40 (0.36, 0.47)	0.40 (0.38, 0.41)	0.40 (0.36, 0.45)	0.40 (0.37, 0.42)	0.39 (0.34, 0.41)
	DL	0.33 (0.30, 0.38)	0.35 (0.29, 0.42)	0.37(0.35, 0.40)	0.36 (0.31, 0.38)	0.35 (0.27, 0.42)

**Table 4:** Cardiovascular variables and dobutamine requirements expressed as median (interquartile range) or mean ± SD in 20 healthy adult horses anesthetized with isoflurane and a of lidocaine (1.3 mg/kg loading dose, followed by 3 mg/kg/hour) with (DL) or without (LIDO) the addition of dexmedetomidine (1.75 µg/kg loading dose, followed by 1.75 µg/kg/hour). HR, heart rate; MAP, mean arterial pressure.

Variable		Baseline	Bolus	30 min	60 min	90 min
HR (beats/min)	LIDO	31 (29, 35)	32.5 (30, 35)	32 (30, 36)	32 (27, 37)	36.5 (30.5, 40)
	DL ^†^	43.5 (39, 49)	33 (30, 37) *	34.5 (30, 40) *	32 (32, 39) *	31.5 (30.5, 39) *
MAP (mmHg)	LIDO	69 (53, 72)	77.5 (68, 83) *	78 (77, 85) *	79 (65, 88) *	84.5 (77, 94.5) *
	DL	70.5 (63, 90)	80.5 (77, 93) *	80 (76, 84) *	80 (74, 81) *	80 (76, 86.5) *
Dobutamine (μg/kg/minute)	LIDO	0.47 ± 0.38	0.34 ± 0.32	0.36 ± 0.31	0.45 ± 0.46	0.45 ± 0.31
	DL ^†^	1.02 ± 0.72	0.47 ± 0.60	0.58 ± 0.51	0.6 ± 0.53	0.79 ± 0.68

* significantly different from baseline (*p* ≤ 0.05). ^†^ significantly different between treatments (*p* ≤ 0.05).

## Data Availability

The original contributions presented in this study are included in the article. Further inquiries can be directed to the corresponding author(s).

## Abbreviations

The following abbreviations are used in this manuscript:

ASA	American Society of Anesthesiologists
BIC	Bayesian information criterion
C_dyn_	Dynamic respiratory system compliance
CI	95% confidence interval
CRI	Constant rate intravenous infusion
C_qstat_	Quasi-static respiratory system compliance
DL	Treatment with 1.75 μg/kg over 15 min, then 1.75 μg/kg/hour
F-shunt	Shunt fraction
Fe′Iso	End-tidal isoflurane concentration
FiO_2_	Fraction of inspired oxygen
fR	Respiratory rate
HR	Heart rate
I:E	Inspiratory-to-expiratory ratio
IV	Endovenous
LIDO	Treatment with lidocaine 1.3 mg/kg over 15 min, then 3 mg/kg/hour
MAP	Mean arterial pressure
Pa-Pe’CO_2_	Arterial to end-tidal CO_2_ difference
PaO_2_	Arterial partial pressure of oxygen
PaO_2_:FiO_2_	Arterial partial pressure over fraction inspired oxygen
P_Atm_	Atmospheric pressure
Pe′CO_2_	End-tidal carbon dioxide tension
P_E_CO_2_	Mixed expired CO_2_ pressure
PEEP	Positive end-expiratory pressure
PIP	Peak inspiratory pressure
P_plat_	Plateau pressure
Q1, Q3	Interquartile range
SD	Standard deviation
SpO_2_	Hemoglobin oxygen saturation
VCap	Volumetric capnography
Ve	Minute ventilation
V_T_	Expiratory tidal volume
